# Green accounting and ESG-driven eco-efficiency in European financial institutions: A two-stage DEA–CRITIC-TOPSIS evaluation

**DOI:** 10.1371/journal.pone.0334882

**Published:** 2025-10-23

**Authors:** George Sklavos, Georgia Zournatzidou, Konstantina Ragazou, Nikolaos Sariannidis

**Affiliations:** 1 Department of Business Administration, University of Thessaly, Larissa, Greece; 2 Department of Business Administration, University of Western Macedonia, Grevena, Greece; 3 Department of Management Science and Technology, University of Western Macedonia, Kozani, Greece; 4 Department of Accounting and Finance, University of Western Macedonia, Kozani, Greece; University of Economics Ho Chi Minh City, VIET NAM

## Abstract

**Background:**

Evaluation of the eco-efficiency of financial institutions and their underlying green accounting practices is imperative as Environmental, Social, and Governance (ESG) principles become ingrained in financial regulation and investment strategy. Nevertheless, the current ESG assessments frequently suffer from a lack of a dual focus on governance quality and performance, which raises concerns about misaligned reporting and Greenwashing.

**Objective:**

This investigation suggests a two-stage methodological framework for evaluating the eco-efficiency of European financial institutions that is driven by ESG and evaluating the impact of internal green accounting practices on sustainability performance.

**Methods:**

Data Envelopment Analysis (DEA) is implemented in the initial phase to calculate eco-efficiency scores that are determined by financial outputs and environmental inputs (GHG emissions, energy consumption, assets). The second stage employs the CRITIC-TOPSIS method to rank 365 institutions according to seven governance-related green accounting criteria. These criteria are derived from the Refinitiv ESG Screener CO₂ dataset. The performance frontiers are identified by DEA, while the contribution of internal sustainability mechanisms is assessed by CRITIC-TOPSIS.

**Results:**

According to the DEA results, only 38% of institutions are entirely efficient, with a substantial degree of variation across the sample. The results of the CRITIC-TOPSIS analysis indicate that the most reliable predictors of green accounting quality are governance indicators, including the presence of an ESG committee and board supervision of climate risks. According to a moderate positive correlation between DEA scores and TOPSIS rankings, eco-efficiency and green accounting maturity are related, but they are not entirely aligned.

**Conclusions:**

The results underscore the importance of integrating institutional governance evaluations with operational performance metrics to accurately evaluate sustainability. Theoretical and methodological contributions to the disciplines of environmental accounting and sustainable finance are made by this integrated framework, which provides regulators, rating agencies, and institutional decision-makers with valuable insights.

## 1. Introduction

The financial sector is essential in the promotion of sustainable development, as it allocates resources to activities that are socially equitable and environmentally responsible. In recent years, financial institutions have faced increasing pressure from regulators, investors, and civil society to incorporate sustainability issues into their operational and strategic frameworks. Consequently, in order to enhance transparency, credibility, and long-term risk management, numerous organizations have implemented Environmental, Social, and Governance (ESG) procedures and reporting requirements [1,2]. Nonetheless, despite the surge in ESG disclosures, apprehensions remain about the efficacy and genuineness of these practices, especially in the context of increasing allegations of greenwashing [3,4].

This transformation is additionally reinforced by regulatory mandates in the European Union, specifically the EU Taxonomy Regulation and the Sustainable Finance Disclosure Regulation (SFDR). Financial market participants are required to disclose the sustainability-related risks and impacts of their investment decisions under the SFDR, while the EU Taxonomy provides a classification system to ascertain whether an economic activity is environmentally sustainable. The objective of these instruments is to standardize ESG evaluation, reduce greenwashing, and improve the accountability of financial institutions. As a result, financial actors are now obligated to align their internal governance systems and disclosure practices with legally mandated sustainability metrics. This has increased the demand for analytical tools that can evaluate the robustness of green accounting frameworks and ESG performance.

ESG reporting frequently fails to accurately represent the internal processes and governance structures that regulate the administration and execution of sustainability initiatives, despite the fact that it conveys performance results. While numerous ESG evaluation frameworks prioritize external measures, such as emissions levels or environmental scores, they fail to adequately address the institutional frameworks that underlie data quality, target-setting, or decision-making processes. As a result, it is imperative to develop analytical frameworks that assess the quality and profundity of green accounting processes within firms and analyze environmental performance outcomes [5–7].

This study proposes a two-stage empirical framework to assess the ESG-driven eco-efficiency of financial institutions and to analyze the structural and procedural factors influencing their environmental performance. The initial phase uses Data Envelopment Analysis (DEA), a prevalent non-parametric method, to evaluate the comparative eco-efficiency of institutions utilizing financial and environmental inputs and outputs. The second step utilizes the CRITIC-TOPSIS technique, a hybrid multi-criteria decision-making methodology, to evaluate and rank institutions according to specific green accounting indicators, including governance supervision, verification systems, and strategic climate strategies.

The primary aims of this research are threefold: (1) to evaluate the comparative eco-efficiency of European financial institutions utilizing DEA; (2) to determine the green accounting practices that most substantially affect sustainability rankings through CRITIC-TOPSIS; and (3) to analyze the correspondence—or absence thereof—between operational efficiency and internal sustainability governance. This study enhances the understanding of how financial institutions can bolster the credibility and effectiveness of their sustainability strategies, while enabling policymakers and stakeholders to distinguish between superficial ESG compliance and genuine environmental commitment.

The subsequent sections of this work are organized as follows: Section 2 examines the pertinent literature on ESG performance, eco-efficiency, and green accounting systems. Section 3 delineates the materials and methodologies, encompassing the DEA and CRITIC-TOPSIS models. Section 4 delineates the empirical findings, whereas Section 5 examines their theoretical and practical ramifications. Section 6 ends the study by summarizing principal results, delineating limitations, and suggesting avenues for further research.

## 2. Literature review

The incorporation of sustainability principles in financial institutions has emerged as a primary concern for academia and regulators, especially due to increasing climate-related risks, sustainable financing requirements, and investor demands for ESG accountability. Environmental, Social, and Governance (ESG) elements have transitioned from optional frameworks to strategic necessities, particularly in the financial industry, where institutional reputation and risk exposure are intricately linked to environmental performance [8–10].

Eco-efficiency, characterized as the capacity to provide favorable outputs while minimizing environmental repercussions, has become an essential criterion for evaluating institutional sustainability [11]. Data Envelopment Analysis (DEA), a non-parametric frontier methodology, has been extensively employed to assess eco-efficiency in financial organizations, such as banks, insurance companies, and asset managers. The methodological flexibility of DEA facilitates the comparison of many inputs and outcomes without presupposing a particular functional form, rendering it particularly appropriates for sustainability situations. Nonetheless, despite its advantages, DEA frequently exhibits a deficiency in diagnostic capability, especially for the institutional factors influencing performance [12–15].

To tackle this issue, researchers have progressively integrated DEA with supplementary analytical techniques that encompass governance, structural, or policy aspects. Multi-Criteria Decision-Making (MCDM) strategies, such the CRITIC (CRiteria Importance Through Intercriteria Correlation) method, have become prominent for their capacity to provide objective weights derived from data variability and dependency [16]. The integration of TOPSIS (Technique for Order Preference by Similarity to Ideal Solution) with the CRITIC technique enables a clear and systematic ranking of decision units according to sustainability-related criteria. Recent implementations of CRITIC-TOPSIS in environmental and social performance evaluations have proven its efficacy in mitigating subjectivity in sustainability assessments, however its use in financial ESG evaluation and green accounting remains constrained [17,18].

The theory of green accounting enhances these methodological advancements by providing a conceptual framework that incorporates environmental factors into financial planning, reporting, and strategic governance. It encompasses carbon accounting, environmental cost analysis, and sustainability auditing methodologies that enable corporations to absorb externalities. In financial organizations, the adoption of green accounting has been inconsistent, frequently influenced by soft law mechanisms such as the Global Reporting Initiative (GRI), the Carbon Disclosure Project (CDP), and, more recently, EU Taxonomy mandates. The voluntary aspect of several frameworks results in discrepancies in disclosure quality and restricts comparison among enterprises and countries [19–21].

Institutional governance is pivotal in determining the legitimacy and profundity of green accounting. Studies indicate that companies with robust board-level ESG governance, established sustainability committees, and verified disclosures are more inclined to generate consistent and credible sustainability reports [22–24]. Concerns with greenwashing—characterized as the deception or embellishment of environmental performance for reputational gain—continue to pervade the finance industry. The disparity between ESG ratings and actual sustainable practices has led researchers to promote integrated models that evaluate both performance outcomes and governance structures [25].

This study enhances the literature by integrating DEA and CRITIC-TOPSIS into a unified framework to assess the ESG-oriented eco-efficiency and green accounting practices of European financial institutions. This dual-stage methodology offers a comprehensive perspective on sustainability, integrating quantitative performance metrics with institutional quality factors, therefore tackling ongoing issues with the openness, correctness, and accountability of ESG assertions within the financial sector.

## 3. Materials and methods

This study utilizes a two-stage empirical framework to assess the eco-efficiency of 365 publicly traded European financial institutions that are driven by ESG and to investigate the influence of green accounting standards on institutional performance variances. This section elucidates the rationale behind the selection of DEA and CRITIC–TOPSIS over other multi-criteria decision-making (MCDM) techniques to enhance methodological transparency and address potential concerns regarding model selection.

The selection of DEA and CRITIC–TOPSIS was influenced by both practical and methodological factors. The relative eco-efficiency of decision-making units (DMUs) can be estimated without the necessity of pre-assigned weights using DEA, a non-parametric technique that has its roots in operations research. When financial institutions with diverse ESG and green accounting profiles are benchmarked, this attribute is particularly advantageous. DEA’s capacity to manage multiple inputs and outputs based on observed data, without subjective assumptions, further enhances its suitability for cross-sectional eco-efficiency analysis.

To improve the evaluation of institutional performance, CRITIC–TOPSIS was implemented in the second stage through the integration of ESG-related disclosure and green accounting indicators. The CRITIC method objectively assigns weights based on the standard deviation of indicators and the correlation structure among them, thereby illustrating the intensity of contrast and inter-criteria conflict. The entropy method’s primary limitation is that it only considers variability without accounting for indicator redundancy. This is addressed by this. In the interim, TOPSIS organizes alternatives in accordance with their proximity to an optimal solution, providing a user-friendly framework for assessing the ESG alignment of institutions. The CRITIC–TOPSIS approach ensures replicability across a large sample and reduces subjectivity, in contrast to methods like AHP, which rely heavily on expert judgment and pairwise comparisons. Therefore, the integration of DEA and CRITIC–TOPSIS enables a comprehensive and exhaustive assessment of the eco-efficiency of ESGs. [17,18,26].

### 3.1 DEA-based assessment of eco-efficiency

Eco-efficiency ratings were calculated with a non-oriented DEA model based on the assumption of Variable Returns to Scale (VRS), executed by MAXDEA 8. The non-oriented paradigm was chosen to facilitate the concurrent assessment of input reduction and output enhancement [27–30]. The VRS model was implemented to address scale variability across financial institutions, hence facilitating comparison between enterprises of varying sizes. The model’s application of the maximum distance to border configuration facilitated improved discriminatory capability among units.

The mathematical formulation of the output-oriented DEA model is as follows:


$${max}_{\theta ,\lambda }\;Subject\;to\;:\;\sum_{j=\text{1}}^{n}\lambda_{j}x_{ij}\leq x_{io},{\;\;\;\;\;\;\;\;\forall }_{i}\sum_{j=\text{1}}^{n}\lambda_{j}{xy}_{rj}\geq {\vartheta y}_{ro},{\;\;\;\;\;\;\forall }_{r}\sum_{j=\text{1}}^{n}\lambda_{j}=\text{1},\;\lambda_{j}\geq \text{0}$$

In this formulation $x_{ij}\;and\;y_{rj}$ represent the input and output values for the $j^{th}$ DMU, $\lambda_{j}\;$are the intensity variables that generate the efficiency frontier ϑ, is the efficiency score, and is the total number of DMUs.

Data were gathered for the fiscal year 2024 via the Refinitiv ESG Screener. Table 1 delineates the input and output variables used in the DEA model. Inputs encompass environmental and resource consumption metrics, whilst outputs denote financial performance and the quality of environmental disclosures.

**Table 1:** DEA input and output variables.

Category	Variable	Description
Input	Total Assets (USD)	Institutional size and resource base
Input	Energy Use (MWh)	Operational energy consumption
Input	Total GHG Emissions (Scope 1, 2, 3; tCO₂e)	Environmental burden
Output	Revenue (USD)	Financial output
Output	Environmental Pillar Score	ESG-based environmental performance indicator
Output	Emissions Score	Quality of emissions reporting and management

DEA efficiency scores vary from 0 to 1, with a score of 1 signifying that an institution is positioned on the efficiency frontier. The scores were the foundation for the subsequent phase of analysis.

### 3.2 Evaluation of green accounting practices using CRITIC-TOPSIS

To assess the impact of green accounting procedures on eco-efficiency, seven criteria were derived using the Refinitiv ESG Screener CO₂ dataset. These metrics signify essential aspects of environmental governance, transparency, and emissions control. Table 2 delineates the chosen criteria.

**Table 2:** Green accounting criteria used in CRITIC-TOPSIS.

Criterion	Description
Total CO₂ Emissions (Scope 1 + 2 + 3)	Consolidated carbon footprint across all scopes
Tax Data Verification (binary)	Indicates the external verification status of tax data
VOC Emissions Reduction (binary)	Denotes measures to mitigate emissions of volatile organic compounds.
Board Oversight of Climate Change Risks (binary)	Indicates board-level oversight of climate matters
Transition Plan for Scope 3 Emissions (binary)	Denotes the existence of a scope 3 reduction approach
Policy on Tax Transparency (binary)	Denotes the presence of a formal tax transparency policy
Governance Board Committee Presence (binary)	Denotes the existence of a sustainability or ESG board committee

All selected criteria were transformed into quantitative format as required and standardized by Min-Max scaling to guarantee comparability among institutions. The employed normalization formula is:


$$x_{ij}^{*}=\frac{x_{ij}-\text{min}(x_{j})}{\text{max}\left(x_{j}\right)-\text{min}(x_{j})}$$

Where $x_{ij}^{*}$ is the normalized score, $x_{ij}$ is the raw score, and $\text{max}\left(x_{j}\right),\text{min}(x_{j})$ denote the maximum and minimum and observed values of criterion.

The next step of the normalization, the CRITIC (CRiteria Importance Through Intercriteria Correlation) approach was employed to calculate objective weights for each criterion. This method evaluates both the standard deviation and the level of discord (i.e., absence of correlation) among criteria to ascertain their significance. The calculation of the weighting is as follows:


$$C_{j}=\sigma_{j}\sum_{k=\text{1}}^{m}\left(\text{1}-r_{jk}\right)and\;w_{j}=\frac{C_{j}}{\sum_{j=\text{1}}^{m}C_{j}}$$

Where $\sigma_{j}$ is the standard deviation of criterion $,r_{jk}$, represents the Pearson correlation coefficient between criteria j and k, and $w_{j}$ is the normalized weight.

Institutional rankings were subsequently established with the Technique for Order Preference by Similarity to Ideal Solution (TOPSIS). The weighted normalized decision matrix was first formulated as follows:


$$v_{ij}=w_{j}x_{ij}^{*}$$

The ideal and anti-ideal solutions were then identified as:


$$A^{+}=\left\{\text{max}(v_{ij}\right\},\;A^{-}=\left\{\text{min}(v_{ij}\right\}$$

The Euclidean distances to the ideal and anti-ideal solutions were subsequently calculated:


$$S_{i}^{+}=\sqrt{\sum {\mathrm{\backslash nolimits}}_{j=\text{1}}^{m}(v_{ij}-A_{j}^{+})²}$$

$$S_{i}^{-}=\sqrt{\sum {\mathrm{\backslash nolimits}}_{j=\text{1}}^{m}(v_{ij}-A_{j}^{-})²}$$

Finally, the relative closeness coefficient was calculated as:


$$C_{i}=\frac{S_{i}^{-}}{S_{i}^{+}+S_{i}^{-}}$$

Institutions with higher $C_{i}$ values exhibit stronger alignment with the ideal green accounting profile. This ranking system allows for a comprehensive comparison of institutional performance in terms of green accounting maturity and supports the identification of those organizations most effectively aligning environmental governance with eco-efficiency outcomes.

## 4. Results

The empirical findings from the application of the two-stage methodological framework described above are presented in this section. Initially, the eco-efficiency levels of the financial institutions under examination are reflected in the results of the DEA analysis. Secondly, the CRITIC-TOPSIS analysis is presented, which evaluates the relative significance of each criterion and ranks institutions according to their green accounting practices. To identify institutional patterns and implications for environmental performance evaluation in the financial sector, a comparative discussion of the two sets of results is included.

### 4.1 Eco-efficiency assessment results (DEA)

The DEA model’s output indicates a significant degree of variation in eco-efficiency among the 365 listed European financial institutions for the fiscal year 2024 (Table 3). The mean efficiency score is 0.743, with a median of 0.808, and the range is from a minimum of 0.351 to a maximum of 1.000. It is important to note that approximately 38% of the institutions attained a score of 1.000, which suggests that they are on the best-practice frontier and are therefore entirely efficient in converting inputs (assets, energy, emissions) into financial and ESG-related outputs.

**Table 3:** Summary statistics of DEA efficiency scores.

Statistic	Value
Minimum	0.351
Maximum	1.000
Mean	0.743
Median	0.808
Standard Deviation	0.192
Number of Efficient DMUs (Score = 1)	139 (≈38%)

**Fig 1:**
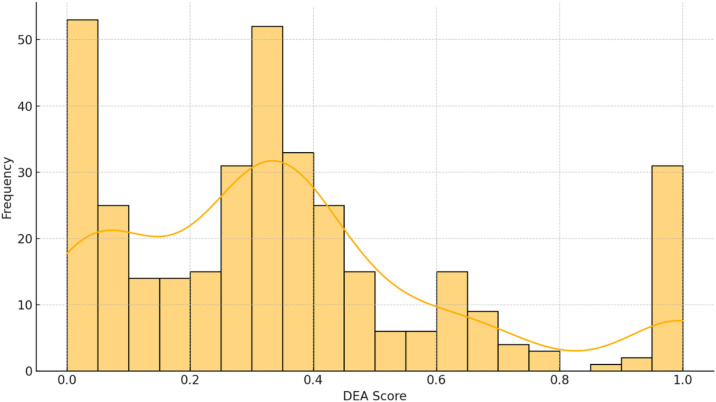
Distribution of DEA eco-efficiency ratings across 364 European financial organizations.

Fig 1 illustrates a histogram of the eco-efficiency scores to improve the interpretability of the DEA findings. The distribution exhibits a left-skewed pattern, with a concentration of financial institutions aggregating around lower to moderate efficiency levels and a smaller subset achieving full efficiency (score = 1.00). The disparity in eco-efficiency performance across the sector is underscored by this visual distribution, which emphasizes the necessity of targeted governance and sustainability reforms.

Thus, the sector’s moderate level of inefficiency is indicated by the distribution of DEA scores, with over half of the institutions scoring below the efficiency frontier (Table 4). These inefficiencies may be the result of suboptimal energy utilization, excess greenhouse gas emissions, or lower scores in emissions transparency and environmental pillar performance. The findings underscore the potential for a substantial portion of the financial sector to enhance both operational sustainability and ESG reporting.

**Table 4:** Distribution of DEA scores by quartile, and number of fully efficient institutions.

Quartile	Score Range	Number of Institutions	Percentage
Q1 (Lowest 25%)	0.351–0.613	91	25.0%
Q2	0.614–0.742	91	25.0%
Q3	0.743–0.999	88	24.1%
Q4 (Fully Efficient)	1.000	95	26.0%
**Total**	**—**	**365**	**100.0%**

These findings emphasize the necessity of more precise sustainability strategies within financial institutions, particularly those that fell below the second quartile of DEA performance.

### 4.2 Evaluation of green accounting performance (CRITIC-TOPSIS)

The CRITIC-TOPSIS research evaluates and ranks the identical institutions according to the quality and comprehensiveness of their green accounting processes. The CRITIC technique objectively assessed the significance of each criterion using statistical dispersion and correlation redundancy. The resultant weights (Table 5) indicate the extent to which each criterion contributes to the distinction of institutions.

**Table 5:** CRITIC weights for all seven criteria.

Criterion	CRITIC Weight
Board Oversight of Climate Change Risks	0.242
Governance Board Committee Presence	0.215
Policy on Tax Transparency	0.211
Tax Data Verification	0.143
Transition Plan for Scope 3 Emissions	0.098
Total CO₂ Emissions (Scope 1 + 2 + 3)	0.061
VOC Emissions Reduction	0.031

The study found the three most significant green accounting indicators as Board Oversight of Climate Change Risks (CRITIC weight: 0.242), Governance Board Committee Presence (0.215), and Policy on Tax Transparency (0.211). 0.211

The findings indicate that institutional governance frameworks and formal transparency procedures significantly influence the disparities in green accounting maturity. Conversely, metrics like VOC Emissions Reduction (0.031) and Total CO₂ Emissions (0.061) shown less discriminatory power among institutions, perhaps attributable to reduced variability or more standardized reporting practices.

TOPSIS scores (Table 6) varied from 0.000 to 0.904, exhibiting a mean of 0.355 and a standard deviation of 0.246. The leading decile of institutions exhibited sophisticated implementation of green accounting standards, encompassing comprehensive Scope 3 transition strategies, validated tax data disclosures, and proactive board-level ESG governance. Conversely, institutions in the lowest decile had a deficiency in these structural and procedural components, indicating a shallow or undeveloped methodology for green accounting.

**Table 6:** TOPSIS ranking results: summary statistics and top 10 and bottom 10 institutions.

Statistic	Value	
Minimum	0.000	
Maximum	0.904	
Mean	0.355	
Median	0.341	
Standard Deviation	0.246	
**Top 10 Institutions by TOPSIS Score**		
**Rank**	**Institution ID**	**TOPSIS Score**
1	DMU_103	0.904
2	DMU_118	0.880
3	DMU_152	0.862
4	DMU_189	0.850
5	DMU_201	0.838
6	DMU_76	0.832
7	DMU_264	0.820
8	DMU_311	0.816
9	DMU_95	0.804
10	DMU_209	0.798
**Bottom 10 Institutions by TOPSIS Score**		
**Rank**	**Institution ID**	**TOPSIS Score**
356	DMU_67	0.051
357	DMU_240	0.043
358	DMU_302	0.036
359	DMU_148	0.029
360	DMU_84	0.026
361	DMU_33	0.020
362	DMU_178	0.015
363	DMU_270	0.011
364	DMU_5	0.006
365	DMU_251	0.000

### 4.3 Comparative analysis of DEA and CRITIC-TOPSIS results

A comparative analysis of DEA and TOPSIS scores was conducted to investigate the relationship between institutional eco-efficiency and green accounting maturity and are provided in S1 Table (Supporting Information). A moderate positive relationship was indicated by a preliminary Pearson correlation coefficient (Table 7) of approximately 0.41 (p < 0.01) between DEA scores and TOPSIS closeness coefficients. This implies that the two assessments are meaningfully related, even though they measure distinct dimensions of sustainable performance.

**Table 7:** Correlation matrix of DEA scores and individual CRITIC-TOPSIS criteria.

Variable	DEA Score
Board Oversight of Climate Change Risks	0.42
Governance Board Committee Presence	0.39
Policy on Tax Transparency	0.35
Tax Data Verification	0.28
Transition Plan for Scope 3 Emissions	0.23
Total CO₂ Emissions (Scope 1 + 2 + 3)	−0.12
VOC Emissions Reduction	−0.08

* All positive correlations are significant at p < 0.01, except where indicated.

Institutions that demonstrated robust performance in both models were more likely to demonstrate a consistent dedication to sustainability, both operationally (low emissions and high financial outputs) and strategically (strong disclosure and governance structures). In contrast, a small number of institutions exhibited high DEA scores but relatively low TOPSIS rankings. This group may demonstrate operational efficiency without a corresponding investment in transparent sustainability governance, a symmetry that may suggest a risk of reputational or greenwashing.

The value of a multidimensional evaluation framework is further emphasized by these findings. CRITIC-TOPSIS offers diagnostic insight into the practices and structures that either support or undermine performance efficiency, while DEA gives a snapshot of performance efficiency. This is from a sustainability accounting perspective. These instruments collectively provide a more comprehensive comprehension of institutional sustainability.

### 4.4. Correlation between DEA and CRITIC–TOPSIS results

**Fig 2:**
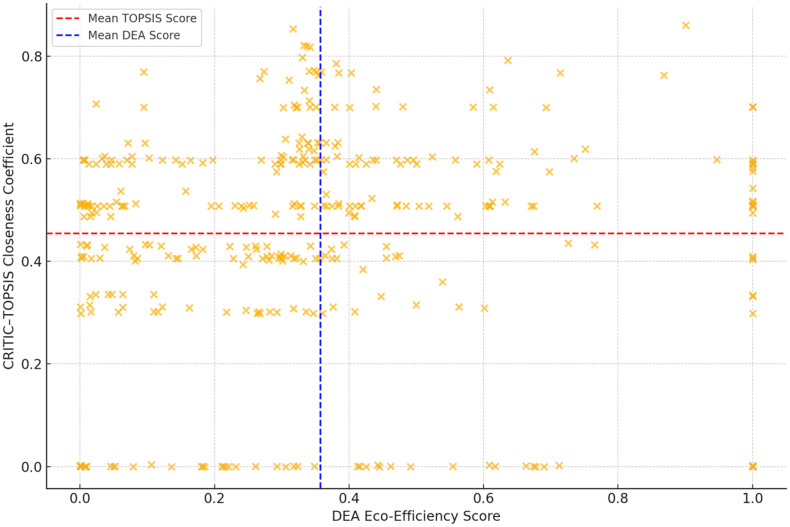
Scatterplot of the DEA vs. CRITIC–TOPSIS scores.

In addition to the numerical correlation results (see Fig 2), a scatterplot was generated to illustrate the correlation between DEA efficiency scores and CRITIC–TOPSIS closeness coefficients across the sample of 364 financial institutions. The quadrant grid, which is based on the mean values of each metric, enables the identification of institutions that exhibit high consistency across both metrics (upper-right quadrant), underperformance on both dimensions (lower-left), or divergence between operational efficiency and ESG disclosure quality (upper-left or lower-right). This visual analysis assists in the identification of performance clusters and outliers that may be obscured when correlation coefficients are employed in isolation.

The Pearson correlation coefficient between DEA scores and CRITIC–TOPSIS closeness coefficients is 0.408, as demonstrated in Table 8. The p-value is less than 0.001, and the 95% confidence interval is [0.326, 0.485]. This moderate positive correlation, which is statistically significant, suggests that institutions with strong eco-efficiency frequently exhibit sound ESG disclosure and governance practices. However, the association is not absolute.

**Table 8:** Pearson correlation of DEA eco-efficiency scores and CRITIC–TOPSIS closeness coefficients.

Statistic	Value
Pearson correlation coefficient	0,408
p-value	< 0.001
95% Confidence Interval (Lower)	0,326
95% Confidence Interval (Upper)	0,485

This result is both theoretically and practically significant. It is confirmation from a theoretical perspective that operational eco-efficiency and green accounting maturity, although they are related, are conceptually distinct constructs. The robustness and comprehensiveness of ESG governance, accounting transparency, and disclosure mechanisms are captured by CRITIC–TOPSIS, while the transmutation of financial and environmental inputs into outputs is reflected in DEA. Consequently, the moderate correlation emphasizes that governance and efficacy do not necessarily evolve in tandem. An institution may demonstrate efficient environmental input-output performance in the absence of mature internal ESG governance systems, or conversely.

This distinction has significant practical implications for stakeholders, including regulators and investors. The moderate correlation is a cautionary tale against overreliance on disclosure-based ESG ratings as proxies for authentic operational performance. It also corroborates the literature’s concerns about potential greenwashing: companies may achieve favorable ESG ratings by optimizing disclosure practices while maintaining suboptimal operational sustainability. As a result, dual-metric evaluations, such as the combined DEA–CRITIC–TOPSIS approach implemented in this study, provide a more comprehensive diagnostic instrument for differentiating between symbolic and substantive ESG strategies.

### 4.5 Analysis of the robustness of CRITIC–TOPSIS rankings

To guarantee the stability and dependability of the CRITIC–TOPSIS model in the presence of data uncertainty, a robustness analysis was implemented by incorporating controlled noise into the normalized ESG indicators. To simulate the variability or potential data inaccuracies that are common in self-reported sustainability disclosures, each ESG input value was perturbed by ±5% using a uniform random distribution. To maintain the complete sample of 364 financial institutions, the median of each indicator was employed to impute missing values in the original dataset.

The perturbed dataset was employed to recalculate the TOPSIS scores, and Spearman’s rank correlation coefficient was employed to evaluate the rank-order correlation between the original and stochastic scores. The correlation was 0.915 (p-value ≈ 1.09 × 10 ⁻ ¹⁴⁴), suggesting that institutional rankings are highly consistent despite fluctuations in input. This illustrates the model’s resilience and bolsters the dependability of the prioritization results that were obtained from the CRITIC–TOPSIS evaluation. The complete comparison between the original and perturbed scores, which includes variations in relative rankings across institutions, is presented in S2 Table (Supporting Information).

## 5. Discussion

This study provides new empirical insights into the eco-efficiency and green accounting practices of European financial institutions. The dual-phase technique, incorporating DEA and CRITIC-TOPSIS, enables a comprehensive viewpoint that transcends traditional evaluations of ESG performance. The identified variability in eco-efficiency among institutions highlights the necessity for focused measures to enhance sustainability results [11,31–33]. The impact of governance-focused green accounting standards on institutional rankings underscores the importance of internal monitoring and openness in determining sustainability pathways [17,18,26].

The DEA findings indicate a significant performance disparity between institutions on the eco-efficiency frontier and those that do not meet this standard. While around 38% of organizations operate at maximum efficiency, the remainder have significant inefficiencies, especially with environmental outputs. This suggests that whereas several institutions have effectively incorporated sustainability into their operational and financial performance, others remain behind, either owing to resource misallocation, inadequate execution of sustainability strategies, or insufficient integration of ESG principles.

The CRITIC-TOPSIS study provides a diagnostic supplement to DEA by pinpointing institutional behaviors that correlate strongly with superior sustainability performance. The emergence of governance-related variables—namely board supervision, ESG committee presence, and tax transparency policy—as the most significant criterion indicates that the structural and procedural integration of ESG is essential. These findings correspond with research highlighting the significance of high-level governance in facilitating the effective execution of sustainability strategies and accountability systems. Notably, more technical environmental indicators, such total CO₂ emissions and VOC reduction, were less significant in differentiating across institutions. This indicates that emissions data, although significant, may inadequately reflect an institution’s green accounting maturity without being contextualized by governance and reporting systems. This also underscores a possible shortcoming in existing ESG grading systems that assign excessive importance to environmental outcomes while neglecting the integrity of the foundational accounting and management frameworks [34–37].

This study’s results indicate a complex relationship between the internal ESG governance structures of financial institutions and eco-efficiency. The DEA results underscore a significant disparity in eco-efficiency, whereas the CRITIC–TOPSIS rankings underscore the critical importance of governance-related green accounting indicators. A correlation analysis was implemented to further investigate the interaction between these dimensions. According to Table 8, the Pearson correlation coefficient between DEA efficiency scores and CRITIC–TOPSIS closeness coefficients was 0.408 (p < 0.001), with a 95% confidence interval spanning from 0.326 to 0.485. This statistically significant and moderate correlation suggests that institutions with robust environmental performance frequently exhibit robust ESG governance practices. The correlation is not sufficiently robust to indicate complete alignment, however.

Operational eco-efficiency and ESG governance are distinct yet interrelated dimensions of sustainable performance, as demonstrated by this partial convergence. It is possible for numerous institutions to accomplish high efficiency in resource utilization without demonstrating comprehensive governance or disclosure mechanisms, and the reverse. This discrepancy may denote potential instances of greenwashing or, conversely, suggest that structural constraints restrict the environmental impact of even well-governed institutions. The findings support the necessity of a dual-assessment framework, as relying exclusively on output-based metrics or governance disclosures may result in a misleading or incomplete understanding of institutional sustainability. The quadrant analysis (Fig 2) adds to this by visualizing clusters of alignment and divergence, providing valuable interpretive insights for investors and regulators.

These findings include several implications. This paper theoretically enhances the literature on sustainable accounting by proposing a dual-method paradigm that combines efficiency analysis with multi-criteria decision-making. This integrated method improves the analytical rigor of sustainability performance evaluations and facilitates the creation of more sophisticated conceptual frameworks that connect governance structures with environmental results. It underscores the necessity of treating ESG not just as external disclosures but as an integral aspect of institutional governance and operational decision-making [38–42].

A number of targeted policy recommendations are derived from the empirical findings of this study, with direct relevance for regulators, financial institutions, investors, and ESG rating agencies. Initially, the pressing necessity for mandatory governance disclosures under regulatory frameworks such as the Sustainable Finance Disclosure Regulation (SFDR) and the Corporate Sustainability Reporting Directive (CSRD) is underscored by the significant impact of governance-related green accounting variables, including the presence of formal sustainability committees, board-level oversight of ESG, and tax transparency policies. Policymakers should broaden the current ESG reporting guidelines to mandate more detailed, verifiable information regarding the structure, frequency, and strategic scope of board supervision on climate and environmental issues. Second, the observed divergence between DEA efficiency scores and CRITIC–TOPSIS rankings in a subset of institutions indicates the possibility of governance decoupling, in which operational sustainability performance is not equivalent to robust internal ESG systems. Latent greenwashing risks may be concealed by this asymmetry. Third-party verification mechanisms, such as standardized assurance procedures for ESG reporting, should be incorporated into disclosure regulations as a corrective measure. These mechanisms should assess governance integrity and procedural robustness in addition to outcomes (e.g., carbon emissions).

Third, the results are in favor of the integration of multi-dimensional evaluation frameworks that combine qualitative, structure-based indicators (e.g., board supervision and ESG strategy) with quantitative outcome-based measures (e.g., DEA-based efficiency) for institutional investors and ESG rating agencies. To discourage the practice of “box-ticking compliance” and promote genuine sustainability transformations, ESG investment ratings and index inclusion criteria should implement dual-performance thresholds that necessitate firms to exhibit both operational eco-efficiency and governance transparency. Finally, the study’s results indicate that even highly eco-efficient firms may underperform in governance metrics, which underscores the necessity of board-level training, strategic alignment of ESG targets with executive compensation, and cross-departmental ESG integration. To align environmental performance with credible green accounting infrastructures, financial institutions should employ sector-specific ESG governance benchmarks and capacity-building initiatives that are supported by regulators and industry bodies.

Notwithstanding its implications, the study had limitations too. The dataset’s cross-sectional nature restricts the capacity to make causal conclusions or evaluate performance longitudinally. Secondly, the research depends on publicly accessible ESG data, which may be prone to self-reporting bias or inadequate disclosures. The methodology presumes universal relevance of chosen criteria across all institutions, perhaps disregarding sector-specific contextual subtleties. The investigation exclusively examines European financial institutions, perhaps constraining the applicability of the findings to other areas or industries.

Subsequent research ought to focus on mitigating these constraints by integrating longitudinal or panel data to evaluate temporal variations in eco-efficiency and governance practices. Comparative analyses between regions or across financial and non-financial sectors may elucidate overarching patterns and context-specific determinants. Additionally, mixed-method techniques that combine quantitative research with comprehensive qualitative case studies may yield a more profound knowledge of the organizational dynamics influencing green accounting adoption and sustainability results.

## 6. Conclusions

This study employs a two-stage analytical framework that integrates the CRITIC–TOPSIS method and Data Envelopment Analysis (DEA) to provide innovative empirical insights into the eco-efficiency and green accounting practices of European financial institutions that are driven by ESG. By combining frontier efficiency measurement with multi-criteria evaluation of governance-related sustainability indicators, the research bridges the divide between the institutional mechanisms that underpin corporate accountability and the environmental performance outcomes.

Only a small number of institutions have achieved efficient frontier, as the results indicate a significant degree of heterogeneity in their eco-efficiency levels. In addition to demonstrating optimal utilization of financial and environmental resources, the most efficient individuals also exhibit comprehensive internal governance structures, particularly in the context of formal sustainability committees and board supervision of climate risks. Beyond mere compliance with disclosure obligations, these results underscore the significance of incorporating ESG considerations into corporate governance.

Additionally, the Pearson correlation analysis between DEA scores and CRITIC–TOPSIS closeness coefficients reveals a statistically significant but moderate association (r = 0.408, p < 0.001), suggesting that operational eco-efficiency and internal ESG governance alignment are closely related but not fully synchronized. This disparity emphasizes the potential for a decoupling between governance maturity and actual performance, which is a critical insight for stakeholders who are concerned about greenwashing. The necessity of integrative assessment frameworks that mutually evaluate institutional disclosure quality and environmental outcomes is thus underscored by the research.

The study makes a theoretical contribution by proposing a hybrid model that is reproducible and improves the robustness of ESG evaluation in intricate organizational situations. Practically, it offers actionable insights for financial institutions, investors, and regulators who are striving to enhance sustainability assessment tools and cultivate credible green accounting systems. However, it is imperative to recognize a few constraints, such as the cross-sectional character of the dataset, the dependance on self-reported ESG metrics, and the limitation to the European financial sector. Research in the future should integrate qualitative assessments of institutional sustainability culture, investigate regional comparisons, and adopt longitudinal designs.

In summary, the holistic DEA–CRITIC–TOPSIS methodology offers a transparent and rigorous instrument for assessing sustainability in the financial sector. This establishes the foundation for a more sophisticated ESG policy design, improved corporate governance standards, and data-driven investment decisions that promote a low-carbon, resilient financial ecosystem.

## Supporting information

S1 Table CRITIC-TOPSIS results. (PDF)

S2 Table Sensitivity of ESG Performance Rankings Using Original and Noisy CRITIC–TOPSIS Scores. (PDF)

## References

[1] Dicuonzo G, Palmaccio M, Shini M. ESG, governance variables and Fintech: An empirical analysis. Research in International Business and Finance. 2024;69:102205. doi: 10.1016/j.ribaf.2023.102205

[2] Ragazou K, Lemonakis C, Passas I, Zopounidis C, Garefalakis A. ESG-driven ecopreneur selection in European financial institutions: entropy and TOPSIS analysis. Management Decision. 2024. doi: 10.1108/MD-12-2023-2425/FULL/XML

[3] Li J. Controlling shareholders’ stock pledges and greenwashing–Evidence from China. Finance Research Letters. 2024;69:106227. doi: 10.1016/j.frl.2024.106227

[4] Pizzetti M, Gatti L, Seele P. Firms Talk, Suppliers Walk: Analyzing the Locus of Greenwashing in the Blame Game and Introducing ‘Vicarious Greenwashing’. J Bus Ethics. 2019;170(1):21–38. doi: 10.1007/s10551-019-04406-2

[5] López-De-Silanes F, Farinha JB, Scannella E. ESG, greenwashing and financial controversies in organizations. https://journals.sagepub.com

[6] Todaro DL, Torelli R. From greenwashing to ESG‐washing: A focus on the circular economy field. Corp Soc Responsibility Env. 2024;31(5):4034–46. doi: 10.1002/csr.2786

[7] Zhao X, Huang X, Liu F, Pan L. Executive power discrepancy and corporate ESG greenwashing. International Review of Financial Analysis. 2024;96:103533. doi: 10.1016/j.irfa.2024.103533

[8] Cardillo MA dos R, Basso LFC. Revisiting knowledge on ESG/CSR and financial performance: A bibliometric and systematic review of moderating variables. Journal of Innovation & Knowledge. 2025;10(1):100648. doi: 10.1016/j.jik.2024.100648

[9] Chen Z, Xie G. ESG disclosure and financial performance: Moderating role of ESG investors. International Review of Financial Analysis. 2022;83:102291. doi: 10.1016/j.irfa.2022.102291

[10] DasGupta R. Financial performance shortfall, ESG controversies, and ESG performance: Evidence from firms around the world. Finance Research Letters. 2022;46:102487. doi: 10.1016/j.frl.2021.102487

[11] WBCSD. Eco-efficiency: creating more value with less impact. 2000.

[12] Madden BJ. Bet on innovation, not Environmental, Social and Governance metrics, to lead the Net Zero transition. Syst Res Behav Sci. 2022;40(3):417–28. doi: 10.1002/sres.2915

[13] Mihaylova-Borisova G, Nenkova P. DEA Efficiency Approach in Comparing Macroeconomic Performance of EU and Balkan Countries. Economic Studies Journal. 2021;(6):42–62.

[14] Shao Q, Yuan J, Lin J, Huang W, Ma J, Ding H. A SBM-DEA based performance evaluation and optimization for social organizations participating in community and home-based elderly care services. PLoS One. 2021;16(3):e0248474. doi: 10.1371/journal.pone.0248474 33730070 PMC7968683

[15] Ullah S, Majeed A, Popp J. Determinants of bank’s efficiency in an emerging economy: A data envelopment analysis approach. PLoS One. 2023;18(3):e0281663. doi: 10.1371/journal.pone.0281663 36917587 PMC10027419

[16] Diakoulaki D, Mavrotas G, Papayannakis L. Determining objective weights in multiple criteria problems: The critic method. Computers & Operations Research. 1995;22(7):763–70. doi: 10.1016/0305-0548(94)00059-h

[17] Hassan I, Alhamrouni I, Azhan NH. A CRITIC–TOPSIS Multi-Criteria Decision-Making Approach for Optimum Site Selection for Solar PV Farm. Energies. 2023;16(10):4245. doi: 10.3390/en16104245

[18] Wang W, Qi Y, Jia B, Yao Y. Dynamic prediction model of spontaneous combustion risk in goaf based on improved CRITIC-G2-TOPSIS method and its application. PLoS One. 2021;16(10):e0257499. doi: 10.1371/journal.pone.0257499 34705831 PMC8550420

[19] Sun Y, Shen Y, Tan Q. The spillover effect of customers’ ESG performance on suppliers’ green innovation quality. China Journal of Accounting Research. 2024;17(3):100362. doi: 10.1016/j.cjar.2024.100362

[20] Xue S, Jiang Y, Wei Q. Green financial accounting and transition in the mining sector in emerging economies. Resources Policy. 2024;89:104683. doi: 10.1016/j.resourpol.2024.104683

[21] Zhang D. Are firms motivated to greenwash by financial constraints? Evidence from global firms’ data. Financ Manag Account. 2022;33(3):459–79. doi: 10.1111/jifm.12153

[22] Bonetti L, Lai A, Stacchezzini R. Stakeholder engagement in the public utility sector: Evidence from Italian ESG reports. Utilities Policy. 2023;84:101649. doi: 10.1016/j.jup.2023.101649

[23] Mishra G, Patro A, Tiwari AK. Does climate governance moderate the relationship between ESG reporting and firm value? Empirical evidence from India. International Review of Economics & Finance. 2024;91:920–41. doi: 10.1016/j.iref.2024.01.059

[24] Mohy-ud-Din K. ESG reporting, corporate green innovation and interaction role of board diversity: A new insight from US. Innovation and Green Development. 2024;3(4):100161. doi: 10.1016/j.igd.2024.100161

[25] Torelli R, Balluchi F, Lazzini A. Greenwashing and environmental communication: Effects on stakeholders’ perceptions. Bus Strat Env. 2019;29(2):407–21. doi: 10.1002/bse.2373

[26] Farid HMA, Dabic-Miletic S, Riaz M, Simic V, Pamucar D. Prioritization of sustainable approaches for smart waste management of automotive fuel cells of road freight vehicles using the q-rung orthopair fuzzy CRITIC-EDAS method. Information Sciences. 2024;661:120162. doi: 10.1016/j.ins.2024.120162

[27] Balcarova T, Pilarova L, Prokop M, Jadrna M, Kvasnickova Stanislavska L, Pilar L. Analysis of green deal communication on twitter: environmental and political perspective. Frontiers in Environmental Science. 2024;12. doi: 10.3389/FENVS.2024.1370568/FULL

[28] Boix-Fayos C, de Vente J. Challenges and potential pathways towards sustainable agriculture within the European Green Deal. Agricultural Systems. 2023;207:103634. doi: 10.1016/j.agsy.2023.103634

[29] Chen Q, Chen S, Liu D. Regret-based cross efficiency evaluation method in a general two-stage DEA system. Computers & Industrial Engineering. 2023;175:108828. doi: 10.1016/j.cie.2022.108828

[30] Yeşilyurt ME, Şahin E, Elbi MD, Kızılkaya A, Koyuncuoğlu MU, Akbaş-Yeşilyurt F. A novel method for computing single output for DEA with application in hospital efficiency. Socio-Economic Planning Sciences. 2021;76:100995. doi: 10.1016/j.seps.2020.100995

[31] Cui Y, Qiu K, Li G, Jiang H, Kong L. Spatiotemporal differentiation of energy eco-efficiency of shipbuilding industry in China. Ocean & Coastal Management. 2022;230:106347. doi: 10.1016/j.ocecoaman.2022.106347

[32] Heikkurinen P, Young CW, Morgan E. Business for sustainable change: Extending eco-efficiency and eco-sufficiency strategies to consumers. Journal of Cleaner Production. 2019;218:656–64. doi: 10.1016/j.jclepro.2019.02.053

[33] Wang F, Wu M, Du X. Does industrial upgrading improve eco-efficiency? Evidence from China’s industrial sector. Energy Economics. 2023;124:106774. doi: 10.1016/j.eneco.2023.106774

[34] Dicuonzo G, Palmaccio M, Shini M. ESG, governance variables and Fintech: An empirical analysis. Research in International Business and Finance. 2024;69:102205. doi: 10.1016/j.ribaf.2023.102205

[35] Huang L. Green bonds and ESG investments: Catalysts for sustainable finance and green economic growth in resource-abundant economies. Resources Policy. 2024;91:104806. doi: 10.1016/j.resourpol.2024.104806

[36] Tong Y, Lau YW, Binti Ngalim SM. Do pilot zones for green finance reform and innovation avoid ESG greenwashing? Evidence from China. Heliyon. 2024;10(13):e33710. doi: 10.1016/j.heliyon.2024.e33710 39044982 PMC11263631

[37] Xie Y. The interactive impact of green finance, ESG performance, and carbon neutrality. Journal of Cleaner Production. 2024;456:142269. doi: 10.1016/j.jclepro.2024.142269

[38] Benuzzi M, Klaser K, Bax K. Which ESG+F dimension matters most to retail investors? An experimental study on financial decisions and future generations. Journal of Behavioral and Experimental Finance. 2024;41:100882. doi: 10.1016/j.jbef.2023.100882

[39] Koemtzopoulos D, Zournatzidou G, Sariannidis N. Can Cryptocurrencies Be Green? The Role of Stablecoins Toward a Carbon Footprint and Sustainable Ecosystem. Sustainability. 2025;17(2):483. doi: 10.3390/su17020483

[40] Lo H-W, Lin S-W. Identifying ESG investment key indicators and selecting investment trust companies by using a Z-fuzzy-based decision-making model. Socio-Economic Planning Sciences. 2023;90:101759. doi: 10.1016/j.seps.2023.101759

[41] Zournatzidou G. Evaluating Executives and Non-Executives’ Impact toward ESG Performance in Banking Sector: An Entropy Weight and TOPSIS Method. Administrative Sciences. 2024;14(10):255. doi: 10.3390/admsci14100255

[42] Zournatzidou G, Mallidis I, Farazakis D, Floros C. Enhancing Bitcoin Price Volatility Estimator Predictions: A Four-Step Methodological Approach Utilizing Elastic Net Regression. Mathematics. 2024;12(9):1392. doi: 10.3390/math12091392

