# Supplementary material for: Green accounting and ESG-driven eco-efficiency in European financial institutions: A two-stage DEA–CRITIC-TOPSIS evaluation
Source: PLoS One. 2025 Oct 23;20(10):e0334882. doi: 10.1371/journal.pone.0334882 (PMC12548880; doi:10.1371/journal.pone.0334882)
Supplement: S1 Table — (PDF) [file pone.0334882.s001.pdf]

Table S1. CRITIC-TOPSIS results

ID	Distance_to_Ideal	Distance_to_Anti_Ideal	TOPSIS_Score	TOPSIS_Rank
1	0,28657	0,16140	0,36030	271
2	0,13003	0,30515	0,70121	32
3	0,12971	0,30516	0,70173	30
4	0,09508	0,31774	0,76968	13
5	0,23021	0,23866	0,50901	150
6	0,18503	0,27540	0,59813	75
7	0,16006	0,28772	0,64255	43
8	0,29881	0,13743	0,31503	282
9	0,29720	0,14777	0,33209	278
10	0,18054	0,27802	0,60628	60
11	0,32716	0,00001	0,00002	325
12	0,27029	0,18741	0,40946	245
13	0,18948	0,27245	0,58981	113
14	0,27362	0,18742	0,40651	248
15	0,30174	0,13006	0,30120	299
16	0,16752	0,28647	0,63100	50
17	0,13015	0,30515	0,70102	34
18	0,16702	0,28648	0,63171	47
19	0,26816	0,18741	0,41137	237
20	0,32878	0,00108	0,00328	308
21	0,16680	0,28647	0,63201	45
23	0,27028	0,18741	0,40947	240
24	0,17512	0,28132	0,61634	58
25	0,09596	0,31773	0,76804	17
26	0,12902	0,30517	0,70285	28
27	0,13016	0,30515	0,70099	36
28	0,12954	0,30515	0,70200	29
29	0,23656	0,23240	0,49557	196
30	0,13002	0,30515	0,70123	31
31	0,18330	0,27608	0,60098	70
32	0,26816	0,18741	0,41137	233
33	0,18522	0,27540	0,59789	82
34	0,29189	0,14777	0,33609	272
35	0,32716	0,00000	0,00000	346
36	0,27393	0,18741	0,40623	255
37	0,18521	0,27540	0,59790	78
38	0,05548	0,32400	0,85381	2
39	0,13016	0,30515	0,70099	35
40	0,32716	0,00000	0,00001	331
41	0,17982	0,27540	0,60498	63
42	0,29189	0,14777	0,33609	273
43	0,13023	0,30515	0,70088	37
44	0,11197	0,31169	0,73571	22
45	0,29720	0,14777	0,33208	280

46	0,32716	0,00000	0,00001	332
47	0,22378	0,23865	0,51608	137
48	0,30093	0,13502	0,30971	288
49	0,18900	0,27245	0,59042	102
50	0,23066	0,23865	0,50852	179
51	0,32716	0,00000	0,00000	343
52	0,33014	0,00024	0,00071	315
53	0,29261	0,14777	0,33555	276
54	0,32716	0,00000	0,00001	334
55	0,11269	0,31167	0,73444	24
56	0,32712	0,00079	0,00240	311
57	0,29189	0,14777	0,33609	274
58	0,29802	0,13497	0,31171	284
59	0,23062	0,23865	0,50856	162
60	0,23063	0,23865	0,50855	166
61	0,09482	0,31773	0,77017	11
62	0,18955	0,27245	0,58971	118
63	0,30211	0,13002	0,30088	301
64	0,07122	0,32399	0,81980	4
65	0,07197	0,32399	0,81825	5
66	0,33190	0,00003	0,00009	319
67	0,32716	0,00000	0,00000	340
68	0,33010	0,00055	0,00168	313
69	0,18956	0,27245	0,58971	121
70	0,26258	0,20013	0,43252	212
71	0,08338	0,31858	0,79256	7
72	0,22632	0,23865	0,51326	142
73	0,32716	0,00000	0,00000	342
74	0,32716	0,00000	0,00000	336
75	0,26816	0,18741	0,41137	236
76	0,18173	0,27495	0,60207	68
77	0,09515	0,31773	0,76954	14
78	0,33174	0,00097	0,00291	309
79	0,12610	0,30515	0,70759	26
80	0,23058	0,23865	0,50861	158
81	0,30021	0,13002	0,30221	292
82	0,30538	0,13002	0,29862	305
83	0,18956	0,27245	0,58971	120
84	0,23066	0,23865	0,50852	180
85	0,23066	0,23865	0,50851	194
86	0,24033	0,22890	0,48781	206
87	0,27367	0,18741	0,40646	249
88	0,27028	0,18741	0,40946	242
89	0,18954	0,27245	0,58973	115
90	0,23042	0,23866	0,50878	153
91	0,23619	0,22890	0,49216	200

92	0,09447	0,31776	0,77083	10
93	0,18457	0,27540	0,59874	72
94	0,33004	0,00094	0,00284	310
95	0,18919	0,27245	0,59018	103
96	0,32716	0,00000	0,00000	351
97	0,16759	0,28647	0,63090	52
98	0,22631	0,24277	0,51755	134
99	0,24514	0,00000	0,00001	329
100	0,23066	0,23865	0,50851	189
101	0,09355	0,31775	0,77254	9
102	0,30021	0,13002	0,30221	295
103	0,23065	0,23865	0,50852	176
104	0,18265	0,27554	0,60137	69
105	0,16749	0,28647	0,63104	48
106	0,10213	0,31287	0,75390	21
107	0,17972	0,27540	0,60511	62
108	0,18522	0,27540	0,59788	85
109	0,32716	0,00000	0,00001	333
110	0,32716	0,00000	0,00000	338
111	0,22971	0,23868	0,50958	148
112	0,17957	0,27540	0,60531	61
113	0,32716	0,00000	0,00000	347
114	0,33018	0,00000	0,00000	345
115	0,27390	0,18741	0,40625	252
116	0,24026	0,22890	0,48789	203
117	0,30537	0,13002	0,29863	304
118	0,18069	0,27590	0,60427	65
119	0,12763	0,30522	0,70514	27
120	0,18153	0,27572	0,60300	67
121	0,33191	0,00000	0,00001	328
122	0,23064	0,23865	0,50854	167
123	0,32716	0,00002	0,00005	321
124	0,23058	0,23865	0,50861	157
125	0,30211	0,13002	0,30088	300
126	0,30491	0,13004	0,29898	302
127	0,18713	0,27258	0,59294	99
128	0,22631	0,23865	0,51327	140
129	0,29802	0,13497	0,31171	283
131	0,29802	0,13497	0,31171	286
132	0,22632	0,23865	0,51326	143
133	0,32716	0,00000	0,00000	344
135	0,20781	0,13002	0,38486	270
136	0,24028	0,22890	0,48787	204
137	0,32716	0,00000	0,00000	341
138	0,09498	0,31773	0,76986	12
139	0,17616	0,28127	0,61489	59

140	0,16968	0,28298	0,62516	54
141	0,16683	0,28647	0,63197	46
142	0,23066	0,23865	0,50852	185
143	0,26478	0,20013	0,43046	218
144	0,26816	0,18741	0,41137	232
146	0,29802	0,13497	0,31171	285
147	0,32715	0,00016	0,00048	316
148	0,23053	0,23866	0,50866	155
149	0,27393	0,18741	0,40622	261
150	0,27393	0,18741	0,40622	260
151	0,24029	0,22890	0,48785	205
152	0,18524	0,27540	0,59786	87
153	0,23922	0,22894	0,48902	201
154	0,27388	0,18741	0,40627	251
155	0,32716	0,00000	0,00001	330
156	0,16579	0,18768	0,53097	132
157	0,18946	0,27245	0,58984	111
158	0,30021	0,13002	0,30221	294
160	0,08093	0,31898	0,79763	6
161	0,13041	0,30515	0,70059	39
162	0,23054	0,23865	0,50865	156
163	0,27392	0,18741	0,40624	253
164	0,18937	0,27245	0,58995	107
165	0,30021	0,13002	0,30221	293
167	0,32716	0,00000	0,00000	349
168	0,18956	0,27245	0,58971	122
169	0,22842	0,23884	0,51115	146
170	0,23268	0,23667	0,50424	195
171	0,30538	0,13002	0,29862	307
172	0,23061	0,23865	0,50857	161
173	0,18955	0,27245	0,58972	116
174	0,23064	0,23865	0,50854	169
175	0,32716	0,00000	0,00000	352
176	0,22620	0,23866	0,51340	139
177	0,18932	0,27245	0,59002	106
178	0,30538	0,13002	0,29862	306
179	0,24514	0,00001	0,00004	322
181	0,32716	0,00000	0,00000	350
182	0,24034	0,22890	0,48781	207
183	0,18112	0,27583	0,60363	66
184	0,23063	0,23865	0,50855	165
185	0,09584	0,31773	0,76827	15
186	0,18926	0,27245	0,59009	105
187	0,24514	0,00002	0,00010	318
188	0,23066	0,23865	0,50852	184
189	0,23064	0,23865	0,50854	170

190	0,18522	0,27540	0,59789	80
191	0,18943	0,27245	0,58987	108
192	0,27393	0,18741	0,40622	258
193	0,13030	0,30515	0,70077	38
194	0,29384	0,14777	0,33461	277
195	0,29993	0,13497	0,31034	287
196	0,09811	0,31639	0,76330	18
197	0,19712	0,26698	0,57527	126
198	0,26261	0,20013	0,43248	214
199	0,27383	0,18741	0,40631	250
200	0,23065	0,23865	0,50852	173
201	0,18945	0,27245	0,58984	110
202	0,19714	0,26698	0,57524	127
203	0,23066	0,23865	0,50852	182
204	0,23066	0,23865	0,50852	177
205	0,32716	0,00000	0,00000	354
206	0,23066	0,23865	0,50851	193
207	0,18524	0,27540	0,59786	88
208	0,18454	0,27541	0,59878	71
209	0,27447	0,18497	0,40260	265
210	0,26298	0,20073	0,43288	211
211	0,13011	0,30515	0,70108	33
212	0,22880	0,23876	0,51065	147
213	0,07572	0,23559	0,75678	20
214	0,23066	0,23865	0,50851	191
215	0,23052	0,23866	0,50867	154
216	0,18524	0,27540	0,59786	89
217	0,24024	0,22890	0,48792	202
218	0,22632	0,23865	0,51326	141
219	0,23065	0,23865	0,50852	175
220	0,22378	0,23865	0,51608	136
221	0,18955	0,27245	0,58971	117
222	0,32716	0,00000	0,00000	348
223	0,27393	0,18741	0,40622	263
224	0,16762	0,28647	0,63087	53
225	0,23065	0,23865	0,50853	172
226	0,23066	0,23865	0,50851	190
227	0,26724	0,19682	0,42413	227
228	0,29720	0,14777	0,33209	279
229	0,26816	0,18741	0,41137	231
230	0,30321	0,13497	0,30802	290
231	0,13055	0,30515	0,70037	41
232	0,32716	0,00001	0,00002	327
233	0,19718	0,26698	0,57520	128
234	0,18924	0,27245	0,59011	104
235	0,16147	0,18741	0,53718	130

236	0,18757	0,27245	0,59226	100
237	0,19205	0,26781	0,58238	123
238	0,18516	0,27540	0,59796	76
239	0,26478	0,20013	0,43047	217
240	0,25870	0,20013	0,43617	208
241	0,32716	0,00000	0,00000	339
242	0,20511	0,13754	0,40141	267
243	0,29881	0,13743	0,31503	281
244	0,27029	0,18741	0,40946	244
245	0,12257	0,30515	0,71343	25
246	0,29189	0,14777	0,33609	275
247	0,16750	0,28647	0,63103	49
249	0,23066	0,23865	0,50851	192
250	0,18953	0,27245	0,58974	114
251	0,26816	0,18741	0,41137	235
253	0,27028	0,18741	0,40946	243
254	0,18522	0,27540	0,59788	83
255	0,17403	0,28260	0,61888	56
256	0,26691	0,18919	0,41480	230
257	0,27361	0,18743	0,40654	247
258	0,26725	0,19682	0,42413	228
259	0,17406	0,28260	0,61885	57
260	0,18956	0,27245	0,58971	119
261	0,26351	0,20180	0,43368	209
262	0,32890	0,00000	0,00000	335
263	0,23696	0,23240	0,49514	197
264	0,23065	0,23865	0,50853	171
265	0,23028	0,23866	0,50893	151
266	0,22338	0,24541	0,52350	133
267	0,09590	0,31773	0,76814	16
268	0,20578	0,13743	0,40043	268
269	0,23061	0,23865	0,50857	160
270	0,26350	0,19682	0,42757	222
271	0,23065	0,23865	0,50852	174
272	0,26132	0,19682	0,42961	219
273	0,26477	0,20013	0,43048	216
274	0,18518	0,27540	0,59794	77
275	0,20740	0,13497	0,39422	269
276	0,18525	0,27540	0,59784	96
277	0,23063	0,23865	0,50855	164
278	0,30021	0,13002	0,30221	297
279	0,22920	0,24006	0,51157	145
280	0,05263	0,32513	0,86068	1
281	0,16160	0,18741	0,53698	131
282	0,23066	0,23865	0,50852	178
283	0,26133	0,19682	0,42960	221

285	0,18525	0,27540	0,59785	94
286	0,11245	0,31168	0,73487	23
287	0,26351	0,20180	0,43368	210
288	0,27238	0,18921	0,40990	239
289	0,27363	0,18517	0,40361	264
290	0,19682	0,26699	0,57564	124
291	0,23019	0,23868	0,50906	149
292	0,17401	0,28260	0,61890	55
293	0,18522	0,27540	0,59788	84
294	0,08659	0,31820	0,78609	8
295	0,27028	0,18741	0,40947	241
296	0,24514	0,00002	0,00008	320
297	0,32716	0,00001	0,00003	323
298	0,23066	0,23865	0,50852	186
299	0,27565	0,18487	0,40144	266
300	0,19682	0,26699	0,57564	125
301	0,07044	0,32401	0,82142	3
302	0,26133	0,19682	0,42960	220
303	0,30021	0,13002	0,30221	298
304	0,26816	0,18741	0,41137	234
305	0,27393	0,18741	0,40623	256
306	0,22484	0,24006	0,51637	135
307	0,23064	0,23865	0,50854	168
308	0,27393	0,18741	0,40622	259
309	0,32716	0,00001	0,00002	324
310	0,18464	0,27540	0,59864	73
311	0,16200	0,28711	0,63929	44
312	0,30021	0,13002	0,30221	296
313	0,18525	0,27540	0,59784	95
314	0,32716	0,00005	0,00016	317
315	0,18947	0,27245	0,58982	112
316	0,23697	0,23240	0,49513	198
317	0,16756	0,28647	0,63094	51
318	0,13053	0,30515	0,70040	40
319	0,27357	0,18742	0,40655	246
320	0,27392	0,18741	0,40624	254
321	0,18521	0,27540	0,59789	79
322	0,18525	0,27540	0,59785	93
323	0,26351	0,19682	0,42757	223
324	0,23066	0,23865	0,50852	187
325	0,26961	0,18838	0,41133	238
326	0,26724	0,19682	0,42413	226
327	0,32716	0,00000	0,00000	353
328	0,15802	0,18741	0,54253	129
329	0,18525	0,27540	0,59785	92
330	0,22378	0,23865	0,51608	138

331	0,18524	0,27540	0,59786	90
332	0,13055	0,30515	0,70037	42
333	0,18567	0,27283	0,59505	98
334	0,26725	0,19682	0,42412	229
335	0,17982	0,27540	0,60498	64
336	0,30132	0,13497	0,30935	289
337	0,23062	0,23865	0,50856	163
338	0,23066	0,23865	0,50852	181
339	0,22840	0,23667	0,50889	152
340	0,26723	0,19682	0,42414	225
341	0,18522	0,27540	0,59789	81
342	0,32890	0,00000	0,00000	337
343	0,23066	0,23865	0,50852	183
344	0,26544	0,19823	0,42753	224
345	0,18945	0,27245	0,58984	109
346	0,29960	0,13142	0,30491	291
347	0,32887	0,00030	0,00091	314
348	0,18840	0,27248	0,59121	101
349	0,09843	0,31697	0,76306	19
350	0,26261	0,20013	0,43248	213
351	0,18453	0,27315	0,59682	97
352	0,23060	0,23865	0,50858	159
353	0,27393	0,18741	0,40622	257
354	0,27393	0,18741	0,40622	262
355	0,26458	0,20013	0,43066	215
356	0,30535	0,13002	0,29864	303
357	0,22632	0,23865	0,51326	144
358	0,18525	0,27540	0,59785	91
359	0,23697	0,23240	0,49513	199
360	0,18496	0,27540	0,59822	74
361	0,23066	0,23865	0,50852	188
362	0,32716	0,00001	0,00002	326
363	0,18523	0,27540	0,59788	86
364	0,24510	0,00049	0,00199	312
