# Supplementary material for: Green accounting and ESG-driven eco-efficiency in European financial institutions: A two-stage DEA–CRITIC-TOPSIS evaluation
Source: PLoS One. 2025 Oct 23;20(10):e0334882. doi: 10.1371/journal.pone.0334882 (PMC12548880; doi:10.1371/journal.pone.0334882)
Supplement: S2 Table — (PDF) [file pone.0334882.s002.pdf]

Table S2. Sensitivity of ESG Performance Rankings Using Original and Noisy CRITIC–TOPSIS Scores

Original_TOPSIS	Noisy_TOPSIS	Difference	Rank_Original	Rank_Noisy	Rank_Difference
0,27450	0,27418	-0,00032	156	159	3
0,37203	0,35931	-0,01272	45	80	35
0,37659	0,37662	0,00003	41	39	-2
0,44684	0,44517	-0,00167	15	18	3
0,27658	0,28208	0,00550	111	101	-10
0,27503	0,26599	-0,00904	127	222	95
0,50231	0,49623	-0,00608	2	4	2
0,00018	0,00017	-0,00001	350	350	0
0,27432	0,28046	0,00614	217	115	-102
0,49537	0,48580	-0,00958	8	8	0
0,00058	0,00056	-0,00003	305	319	14
0,00015	0,00015	0,00000	354	352	-2
0,36723	0,37276	0,00553	68	42	-26
0,02779	0,02724	-0,00055	261	261	0
0,04931	0,04904	-0,00027	257	257	0
0,43730	0,43169	-0,00561	29	33	4
0,37099	0,37024	-0,00075	51	49	-2
0,44666	0,44751	0,00085	16	17	1
0,00057	0,00054	-0,00002	318	333	15
0,01734	0,01660	-0,00073	263	263	0
0,44489	0,44199	-0,00290	19	21	2
0,27437	0,27697	0,00259	187	138	-49
0,00133	0,00134	0,00001	279	280	1
0,38474	0,37631	-0,00843	38	40	2
0,43665	0,42944	-0,00721	34	35	1
0,37140	0,37026	-0,00114	48	48	0
0,37024	0,36521	-0,00503	53	65	12
0,37093	0,36930	-0,00163	52	54	2
0,01451	0,01435	-0,00016	266	265	-1
0,37266	0,36819	-0,00448	43	57	14
0,28504	0,28268	-0,00236	101	99	-2
0,00084	0,00080	-0,00004	290	290	0
0,27466	0,26167	-0,01299	139	251	112
0,27437	0,28052	0,00616	193	112	-81
0,00057	0,00055	-0,00002	330	324	-6
0,00132	0,00130	-0,00002	281	281	0
0,27474	0,27175	-0,00299	134	182	48
0,44469	0,44127	-0,00342	20	22	2
0,36970	0,36610	-0,00360	56	62	6
0,00057	0,00056	-0,00001	312	313	1
0,27431	0,27474	0,00043	234	156	-78
0,27436	0,27666	0,00230	195	140	-55

0,36718	0,35880	-0,00839	69	84	15
0,37319	0,36690	-0,00628	42	60	18
0,27430	0,26896	-0,00534	249	205	-44
0,00057	0,00056	-0,00001	314	310	-4
0,27437	0,26595	-0,00841	190	223	33
0,06269	0,06315	0,00046	256	256	0
0,36942	0,36081	-0,00862	57	77	20
0,27431	0,26134	-0,01297	227	254	27
0,00057	0,00055	-0,00002	326	332	6
0,00385	0,00380	-0,00005	274	274	0
0,27433	0,26932	-0,00501	215	199	-16
0,00057	0,00058	0,00001	316	306	-10
0,37140	0,37028	-0,00112	49	47	-2
0,01471	0,01422	-0,00049	265	266	1
0,27436	0,28001	0,00565	196	119	-77
0,00059	0,00058	0,00000	303	301	-2
0,27450	0,27135	-0,00315	157	187	30
0,27445	0,28094	0,00649	163	105	-58
0,44108	0,44344	0,00236	24	20	-4
0,36612	0,36157	-0,00456	83	76	-7
0,00015	0,00015	0,00000	351	351	0
0,44212	0,43619	-0,00592	23	27	4
0,43694	0,43953	0,00259	32	24	-8
0,00050	0,00048	-0,00002	343	343	0
0,00057	0,00054	-0,00003	322	340	18
0,00444	0,00454	0,00010	273	272	-1
0,36607	0,36235	-0,00371	86	71	-15
0,27481	0,27403	-0,00078	129	160	31
0,49595	0,50435	0,00839	7	3	-4
0,27431	0,27998	0,00567	232	120	-112
0,00057	0,00056	-0,00001	324	315	-9
0,00057	0,00053	-0,00003	319	341	22
0,00057	0,00055	-0,00002	311	320	9
0,44904	0,44111	-0,00793	14	23	9
0,44338	0,43448	-0,00890	21	30	9
0,00836	0,00834	-0,00002	269	269	0
0,36611	0,35530	-0,01081	84	89	5
0,27464	0,27323	-0,00142	141	171	30
0,00137	0,00139	0,00002	278	278	0
0,00045	0,00043	-0,00002	344	344	0
0,36608	0,35629	-0,00979	85	88	3
0,27434	0,26914	-0,00521	208	202	-6
0,27429	0,26325	-0,01104	255	240	-15
0,27450	0,27395	-0,00055	155	163	8
0,01075	0,01031	-0,00044	268	268	0
0,00085	0,00080	-0,00005	289	291	2

0,36642	0,36064	-0,00578	74	78	4
0,27537	0,26913	-0,00625	122	203	81
0,27431	0,28064	0,00633	224	110	-114
0,45626	0,45470	-0,00157	12	12	0
0,27806	0,26453	-0,01353	106	231	125
0,01504	0,01494	-0,00010	264	264	0
0,36773	0,36010	-0,00764	61	79	18
0,00057	0,00055	-0,00001	335	322	-13
0,43712	0,43543	-0,00168	31	28	-3
0,27452	0,27806	0,00354	152	127	-25
0,27437	0,27253	-0,00184	189	178	-11
0,27430	0,26610	-0,00820	244	220	-24
0,44603	0,44969	0,00366	17	15	-2
0,00084	0,00084	0,00000	291	289	-2
0,27440	0,26971	-0,00469	179	194	15
0,28811	0,28736	-0,00075	100	97	-3
0,43839	0,43390	-0,00449	26	32	6
0,43584	0,43536	-0,00048	35	29	-6
0,27545	0,28046	0,00501	121	114	-7
0,27446	0,26690	-0,00755	162	217	55
0,00057	0,00055	-0,00002	315	331	16
0,00057	0,00055	-0,00002	320	329	9
0,27926	0,28150	0,00224	105	102	-3
0,27724	0,27630	-0,00094	108	142	34
0,00057	0,00055	-0,00001	331	321	-10
0,00055	0,00053	-0,00002	342	342	0
0,00340	0,00338	-0,00002	275	275	0
0,27467	0,26213	-0,01254	136	245	109
0,00100	0,00098	-0,00002	286	286	0
0,30688	0,29526	-0,01161	93	95	2
0,37784	0,37771	-0,00013	40	38	-2
0,29738	0,30032	0,00294	95	94	-1
0,00007	0,00007	0,00000	360	360	0
0,27439	0,28098	0,00659	181	104	-77
0,00065	0,00063	-0,00002	297	297	0
0,27464	0,26158	-0,01307	140	252	112
0,00038	0,00038	-0,00001	345	345	0
0,02041	0,01977	-0,00065	262	262	0
0,37987	0,37020	-0,00967	39	50	11
0,27442	0,27399	-0,00043	171	161	-10
0,00059	0,00058	-0,00001	302	304	2
0,27440	0,26369	-0,01071	177	237	60
0,00057	0,00056	-0,00001	327	312	-15
0,27431	0,27749	0,00318	235	132	-103
0,00057	0,00057	0,00000	328	309	-19
0,00057	0,00054	-0,00003	317	339	22

0,27438	0,27395	-0,00043	183	162	-21
0,27463	0,27540	0,00077	142	154	12
0,00057	0,00055	-0,00002	323	328	5
0,44538	0,44882	0,00344	18	16	-2
0,36663	0,36249	-0,00414	72	70	-2
0,30183	0,30545	0,00362	94	93	-1
0,44228	0,45056	0,00828	22	13	-9
0,27430	0,27385	-0,00045	245	165	-80
0,27429	0,26550	-0,00879	251	226	-25
0,00099	0,00094	-0,00005	287	287	0
0,00057	0,00057	0,00000	337	308	-29
0,00057	0,00055	-0,00002	325	325	0
0,00304	0,00306	0,00002	276	276	0
0,27578	0,27581	0,00002	118	145	27
0,00010	0,00010	0,00000	357	357	0
0,00008	0,00008	0,00000	358	358	0
0,27522	0,27820	0,00298	125	126	1
0,27438	0,27370	-0,00068	185	166	-19
0,28116	0,27031	-0,01085	104	192	88
0,00790	0,00781	-0,00009	270	270	0
0,00057	0,00054	-0,00003	310	338	28
0,31959	0,31704	-0,00255	91	92	1
0,36782	0,36200	-0,00582	60	74	14
0,00086	0,00085	-0,00001	288	288	0
0,37215	0,36334	-0,00881	44	69	25
0,51713	0,52123	0,00410	1	1	0
0,36748	0,35861	-0,00887	63	85	22
0,27479	0,26199	-0,01280	130	248	118
0,00079	0,00077	-0,00002	293	293	0
0,36689	0,36171	-0,00518	70	75	5
0,00100	0,00098	-0,00002	285	285	0
0,27431	0,28015	0,00584	226	118	-108
0,00057	0,00055	-0,00001	333	323	-10
0,36605	0,37111	0,00506	89	45	-44
0,29001	0,28235	-0,00766	98	100	2
0,36632	0,36911	0,00279	78	56	-22
0,00008	0,00008	0,00000	359	359	0
0,27459	0,26429	-0,01030	148	233	85
0,36626	0,36749	0,00124	79	59	-20
0,27461	0,26472	-0,00989	146	229	83
0,00057	0,00058	0,00001	336	303	-33
0,27629	0,26524	-0,01106	113	228	115
0,37002	0,35922	-0,01080	54	81	27
0,00024	0,00023	-0,00001	347	347	0
0,27438	0,26912	-0,00526	184	204	20
0,00057	0,00056	-0,00001	308	314	6

0,00057	0,00055	-0,00002	334	327	-7
0,27443	0,27335	-0,00108	169	170	1
0,46538	0,46768	0,00229	11	10	-1
0,27477	0,26176	-0,01300	131	250	119
0,43804	0,43883	0,00079	27	25	-2
0,37114	0,37216	0,00102	50	44	-6
0,27440	0,28066	0,00627	178	109	-69
0,27430	0,26552	-0,00878	242	225	-17
0,27452	0,26214	-0,01238	151	244	93
0,27466	0,26779	-0,00687	137	208	71
0,36810	0,36213	-0,00597	59	73	14
0,00021	0,00021	0,00000	348	348	0
0,36838	0,35787	-0,01051	58	87	29
0,27430	0,27712	0,00281	238	136	-102
0,00030	0,00030	0,00000	346	346	0
0,49756	0,49183	-0,00573	5	5	0
0,27491	0,26239	-0,01253	128	243	115
0,27435	0,27551	0,00116	206	149	-57
0,01236	0,01234	-0,00002	267	267	0
0,27443	0,27938	0,00495	167	121	-46
0,36742	0,36215	-0,00527	65	72	7
0,27466	0,27894	0,00428	138	122	-16
0,27433	0,27127	-0,00306	213	189	-24
0,27435	0,26731	-0,00705	205	212	7
0,00057	0,00054	-0,00003	339	336	-3
0,27429	0,27699	0,00270	253	137	-116
0,27444	0,27464	0,00020	166	157	-9
0,27689	0,27564	-0,00125	109	146	37
0,28222	0,28414	0,00192	102	98	-4
0,33602	0,32273	-0,01329	90	90	0
0,36759	0,36502	-0,00256	62	66	4
0,28869	0,27863	-0,01006	99	124	25
0,50003	0,49056	-0,00946	4	7	3
0,27430	0,26698	-0,00732	248	216	-32
0,27654	0,26726	-0,00928	112	213	101
0,27436	0,26688	-0,00748	203	218	15
0,27593	0,26439	-0,01154	115	232	117
0,27433	0,26588	-0,00845	214	224	10
0,27443	0,27353	-0,00090	168	168	0
0,27439	0,26531	-0,00908	182	227	45
0,36615	0,36995	0,00380	81	51	-30
0,00057	0,00056	-0,00001	332	311	-21
0,00001	0,00001	0,00000	364	364	0
0,43669	0,43720	0,00051	33	26	-7
0,27433	0,27777	0,00343	212	129	-83
0,27430	0,27115	-0,00314	246	190	-56

0,27436	0,27755	0,00319	201	131	-70
0,27430	0,26411	-0,01020	237	236	-1
0,00133	0,00134	0,00001	280	279	-1
0,00056	0,00054	-0,00001	341	334	-7
0,36619	0,36487	-0,00132	80	67	-13
0,00058	0,00055	-0,00003	307	326	19
0,27434	0,27022	-0,00412	210	193	-17
0,36748	0,37062	0,00315	64	46	-18
0,27590	0,27460	-0,00130	116	158	42
0,36639	0,36404	-0,00235	76	68	-8
0,31859	0,31782	-0,00077	92	91	-1
0,27458	0,27163	-0,00294	149	184	35
0,27431	0,26241	-0,01191	223	242	19
0,27727	0,26919	-0,00809	107	201	94
0,00057	0,00054	-0,00003	321	335	14
0,29248	0,28130	-0,01119	96	103	7
0,00070	0,00068	-0,00002	294	294	0
0,00019	0,00018	-0,00001	349	349	0
0,36736	0,35917	-0,00819	66	83	17
0,27436	0,26415	-0,01021	198	235	37
0,43858	0,43089	-0,00769	25	34	9
0,27431	0,26707	-0,00724	222	215	-7
0,27429	0,26453	-0,00977	250	230	-20
0,36642	0,36562	-0,00080	75	64	-11
0,00064	0,00063	-0,00001	298	296	-2
0,00057	0,00056	-0,00002	309	318	9
0,00057	0,00056	-0,00001	340	317	-23
0,27461	0,27282	-0,00179	145	174	29
0,27441	0,28060	0,00619	174	111	-63
0,00101	0,00099	-0,00002	284	284	0
0,04554	0,04621	0,00067	258	258	0
0,27433	0,27249	-0,00184	216	179	-37
0,27441	0,26939	-0,00502	175	196	21
0,36606	0,36952	0,00346	87	52	-35
0,27431	0,28029	0,00598	228	117	-111
0,00015	0,00015	-0,00001	352	353	1
0,00060	0,00059	-0,00001	300	299	-1
0,27446	0,26205	-0,01241	161	247	86
0,27596	0,26735	-0,00862	114	210	96
0,36732	0,36947	0,00215	67	53	-14
0,43719	0,43439	-0,00280	30	31	1
0,27445	0,26207	-0,01238	165	246	81
0,27451	0,27553	0,00102	153	147	-6
0,27441	0,26615	-0,00825	176	219	43
0,27434	0,26944	-0,00490	211	195	-16
0,27462	0,26157	-0,01305	143	253	110

0,27449	0,27268	-0,00181	158	177	19
0,27474	0,28048	0,00574	133	113	-20
0,27454	0,26180	-0,01274	150	249	99
0,27429	0,27173	-0,00256	254	183	-71
0,27451	0,27725	0,00274	154	135	-19
0,00057	0,00055	-0,00002	313	330	17
0,27436	0,27284	-0,00152	200	173	-27
0,49741	0,49088	-0,00653	6	6	0
0,27435	0,27662	0,00226	204	141	-63
0,27432	0,26759	-0,00673	219	209	-10
0,27436	0,27540	0,00104	197	153	-44
0,27581	0,26925	-0,00655	117	200	83
0,27430	0,27742	0,00312	243	133	-110
0,37167	0,37480	0,00314	47	41	-6
0,27434	0,27092	-0,00343	209	191	-18
0,03653	0,03527	-0,00125	259	259	0
0,29108	0,29445	0,00337	97	96	-1
0,27563	0,26725	-0,00838	120	214	94
0,28128	0,27338	-0,00790	103	169	66
0,27447	0,28087	0,00641	160	108	-52
0,27461	0,27389	-0,00072	144	164	20
0,47300	0,46220	-0,01079	10	11	1
0,00129	0,00125	-0,00004	282	282	0
0,27439	0,28091	0,00651	180	106	-74
0,00061	0,00058	-0,00002	299	302	3
0,27432	0,26343	-0,01089	220	238	18
0,27430	0,27731	0,00300	241	134	-107
0,27568	0,27293	-0,00274	119	172	53
0,45151	0,45043	-0,00108	13	14	1
0,27438	0,26932	-0,00505	186	198	12
0,00057	0,00054	-0,00003	329	337	8
0,00065	0,00062	-0,00003	296	298	2
0,00066	0,00065	-0,00001	295	295	0
0,27431	0,27544	0,00114	233	152	-81
0,27448	0,27281	-0,00168	159	175	16
0,00014	0,00013	-0,00001	355	355	0
0,00059	0,00056	-0,00003	304	316	12
0,27662	0,27143	-0,00519	110	185	75
0,47686	0,48292	0,00606	9	9	0
0,00059	0,00059	-0,00001	301	300	-1
0,27429	0,27619	0,00190	252	143	-109
0,00113	0,00113	0,00001	283	283	0
0,36682	0,35920	-0,00762	71	82	11
0,00005	0,00004	0,00000	361	361	0
0,43768	0,44468	0,00700	28	19	-9
0,36635	0,36575	-0,00061	77	63	-14

0,02905	0,02899	-0,00006	260	260	0
0,00244	0,00239	-0,00005	277	277	0
0,27476	0,26326	-0,01151	132	239	107
0,27432	0,27864	0,00432	221	123	-98
0,27432	0,27545	0,00113	218	151	-67
0,27431	0,26312	-0,01119	230	241	11
0,27435	0,27538	0,00103	207	155	-52
0,27443	0,27550	0,00107	170	150	-20
0,00057	0,00058	0,00001	338	305	-33
0,27437	0,28088	0,00651	191	107	-84
0,27431	0,27793	0,00362	229	128	-101
0,27437	0,26937	-0,00500	192	197	5
0,27436	0,27198	-0,00238	202	180	-22
0,36614	0,36631	0,00017	82	61	-21
0,39202	0,38907	-0,00295	37	37	0
0,27430	0,27127	-0,00303	247	188	-59
0,27431	0,27270	-0,00160	236	176	-60
0,00079	0,00078	-0,00001	292	292	0
0,27445	0,26126	-0,01319	164	255	91
0,27431	0,27582	0,00151	231	144	-87
0,36605	0,36922	0,00317	88	55	-33
0,27459	0,26600	-0,00859	147	221	74
0,27442	0,27186	-0,00256	173	181	8
0,00015	0,00014	-0,00001	353	354	1
0,27431	0,27773	0,00342	225	130	-95
0,36999	0,35823	-0,01175	55	86	31
0,36653	0,36819	0,00165	73	58	-15
0,27436	0,27136	-0,00301	194	186	-8
0,00448	0,00433	-0,00015	272	273	1
0,37182	0,37222	0,00040	46	43	-3
0,50035	0,50545	0,00510	3	2	-1
0,27437	0,27822	0,00384	188	125	-63
0,40373	0,40437	0,00064	36	36	0
0,27469	0,27551	0,00082	135	148	13
0,00011	0,00011	0,00000	356	356	0
0,00003	0,00003	0,00000	362	362	0
0,27529	0,26815	-0,00714	123	207	84
0,00528	0,00524	-0,00003	271	271	0
0,27430	0,26889	-0,00542	239	206	-33
0,27436	0,26734	-0,00702	199	211	12
0,00003	0,00003	0,00000	363	363	0
0,27528	0,27362	-0,00166	124	167	43
0,27430	0,28031	0,00601	240	116	-124
0,00058	0,00057	-0,00001	306	307	1
0,27442	0,27695	0,00253	172	139	-33
0,27517	0,26421	-0,01095	126	234	108
